# Macrophages, Inflammation, and Tumor Suppressors: ARF, a New Player in the Game

**DOI:** 10.1155/2012/568783

**Published:** 2012-12-18

**Authors:** Paqui G. Través, Alfonso Luque, Sonsoles Hortelano

**Affiliations:** ^1^Molecular Neurobiology Laboratory, The Salk Institute, 10010 North Torrey Pines Road, San Diego, CA 92037, USA; ^2^Instituto Nacional de Investigación Agraria y Alimentaria (INIA), Centro de Investigación en Sanidad Animal (CISA), Ctra. de Algete a El Casar s/n, Valdeolmos, 28130 Madrid, Spain; ^3^Unidad de Inflamación y Cáncer, Área de Biología Celular y Desarrollo, Centro Nacional de Microbiología, Instituto de Salud Carlos III, Carretera Majadahonda-Pozuelo, Km 2,200, Majadahonda, 28220 Madrid, Spain

## Abstract

The interaction between tumor progression and innate immune system has been well established in the last years. Indeed, several lines of clinical evidence indicate that immune cells such as tumor-associated macrophages (TAMs) interact with tumor cells, favoring growth, angiogenesis, and metastasis of a variety of cancers. In most tumors, TAMs show properties of an alternative polarization phenotype (M2) characterized by the expression of a series of chemokines, cytokines, and proteases that promote immunosuppression, tumor proliferation, and spreading of the cancer cells. Tumor suppressor genes have been traditionally linked to the regulation of cancer progression; however, a growing body of evidence indicates that these genes also play essential roles in the regulation of innate immunity pathways through molecular mechanisms that are still poorly understood. In this paper, we provide an overview of the immunobiology of TAMs as well as what is known about tumor suppressors in the context of immune responses. Recent advances regarding the role of the tumor suppressor ARF as a regulator of inflammation and macrophage polarization are also reviewed.

## 1. Introduction

Immune system constitutes one of the first-line defenses to prevent tumor development due to its ability to identify and destroy tumor cells. This process defined as cancer immunosurveillance was initially described by Ehrlich and subsequently revisited by Thomas and Burnet [1–3], gaining considerable attention in last years. Compelling evidence that immune system modulates cancer has emerged over the last decade from gene-targeted mice studies. Mice deficient in several immune effector cells and molecules including interferon (IFN)-*γ* receptor or signal transducer and activator of transcription 1 (STAT1) [4], natural killer (NK) cells, NK-T cells [5, 6], *γδ* T cells [7, 8], IL-12 [9], perforin [10], and granulocyte-macrophage colony stimulating factor (GM-CSF) [11] have been demonstrated to be more susceptible to tumor development. Collectively, these studies strongly support the concept that the immune response is essential in the development of tumors.

## 2. Macrophages: Key Immune Cells

Macrophages are one of the major widely distributed innate immune cells and present essential roles in the primary response to pathogens, maintenance of tissue homeostasis, inflammation, and immunity. 

Macrophages are derived from bone marrow progenitors as immature monocytes. After circulating in the blood stream, monocytes migrate into tissues where they differentiate into resident macrophages [12].

**Figure 1:**
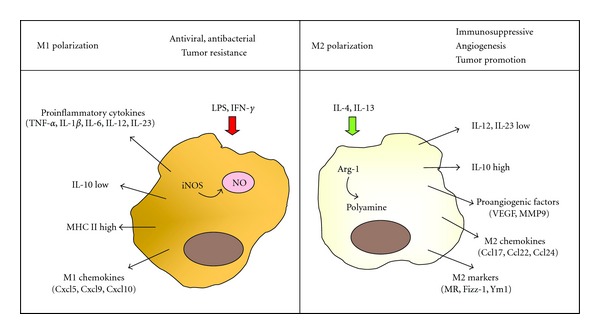
Simplified scheme for M1 and M2 activation of macrophages. M1 macrophages are induced by LPS or IFN-*γ* secreting high levels of classical proinflammatory cytokines such as TNF-*α*, IL-1, IL-6, IL-12, or IL-23, chemokines (e.g., Cxcl9, Cxcl10, and Cxcl5) and increasing their concentrations of NO. In addition, they express high levels of MHC I. IL-4/IL-13 stimulation induces M2 macrophages that downregulate IL-12 and IL-23 expression, release Ccl17, Ccl22 and Ccl24 chemokines and proangiogenic factors, and show increased expression of IL-10. Additionally, they are characterized by expression of MR, Fizz-1, and Ym1.

Macrophages are dynamic cells that might modify their functional profiles in response to a variety of stimuli polarizing to functionally different phenotypes. Two distinct subsets of macrophages have been proposed, including classically activated (M1) and alternatively activated (M2) macrophages [13] (Figure 1). M1 macrophages are induced by IFN-*γ* either alone or cooperating with microbial stimuli such as lipopolysaccharide (LPS) or cytokines (e.g., tumor necrosis factor (TNF)-*α* and GM-CSF). These cells secrete high levels of classical proinflammatory cytokines such as TNF-*α*, interleukin (IL)-1, IL-6, IL-12, or IL-23 and increase their concentrations of nitric oxide (NO), superoxide anions, and oxygen radicals [14, 15]. Furthermore, M1 macrophages can express high levels of major histocompatibility complex (MHC) I and class II antigens and secrete complement factors that facilitate complement-mediated phagocytosis [16]. 

In contrast, IL-4/IL-13 stimulation induces M2 macrophages that reduce IL-12 and IL-23 expression while upregulate the anti-inflammatory cytokine IL-10 [17, 18]. Additionally, they are characterized by the expression of the scavenger receptors mannose receptor (MR) [13], stabilin-1 [19], CD163 [20], and some genes involved in tissue remodelling such as Found in Inflammatory Zone 1 (Fizz-1) and chitinase 3-like 3 (Ym1) [21]. 

An alternative metabolic pathway of L-arginine, catalyzed by arginase-1 (Arg-1) provides another feature of distinction among the two macrophage activation states. M1 macrophages upregulate iNOS to catabolize L-arginine to NO and citrulline, but M2 macrophages are characterized by high expression of Arg-1, a cytosolic enzyme which metabolizes L-arginine to ornithine and polyamines, which are precursors for collagen synthesis and cellular proliferation [22]. 

M1 and M2 macrophages also express a different chemokine repertoire. M1 macrophages produce proinflammatory chemokines such as (C-X-C motif) ligand 9 (Cxcl9), Cxcl10, and Cxcl5 [16], whereas M2 polarization is accompanied by production of Ccl17, Ccl22, and Ccl24 [16].

This plethora of molecules and genes leads macrophages to display distinct functions in virtue to their polarization state. Thus, classically activated macrophages are vital components in the initiation and maintenance of inflammation, as well as in host defense and priming antitumor immune response [13]. For instance, the IL-12 produced by M1 macrophages promotes the differentiation of naive T cells into Th1 cells, stimulates growth of both T and NK cells, and increases bactericidal activity of phagocytes. Moreover, IL-12 exerts an antiangiogenic activity through the increment of the chemokine inducible protein-10 (IP-10 or Cxcl10) [23]. Additionally, M1 chemokines such as Cxcl9, Cxcl10, and Cxcl5 induce the recruitment of Th1 cells, Tc1 cells, and NK cells [16].

 Opposite, M2 macrophages have poor antigen-presenting capability, produce factors that suppress T-cell proliferation and activity, and mainly participate in parasite clearance, tissue remodeling, immune modulation, and tumor progression [15]. Thus, IL-10 expressed by M2 macrophages promotes the production of IL-4 and IL-13 by Th2 cells [24], inhibits the synthesis of proinflammatory cytokines such as IFN-*γ*, IL-2, IL-3, TNF-*α* and GM-CSF, and also suppresses the antigen-presentation capacity of antigen presenting cells. Furthermore, Ccl17, Ccl22, and Ccl24 production favors the attraction of immune-inhibitory cells such as regulatory T-cells (Treg) [25]. 

## 3. Macrophages and Tumor Microenvironment

Compelling evidence has emerged in recent years for macrophages playing an important function in tumor development. Although the role of macrophages in tumors is still controversial, in most human cancers such as breast, prostate, ovarian, cervical, lung carcinoma, and cutaneous melanoma, a macrophage-rich microenvironment has been correlated with a poor prognosis [26, 27]. These tumor-associated macrophages (TAMs) share many common features with the alternatively activated macrophages, showing a typical M2 marker profile with high expression of C-type lectin receptors, stabilin-1, and Arg-1 [25].

Among the cell surface molecules expressed by TAMs, several members of structurally related C-type lectin receptors such as MR and Macrophage galactose-type C-type lectin 1/2 (Mgl-1/2) are included [28]. The MR is an endocytic and phagocytic receptor that was initially described as a bridge between innate immunity and homeostasis [29] due to its ability to bind carbohydrate moieties on several pathogens such as bacteria, fungi, parasites, and viruses. Mgl-1/2 are induced on macrophages by parasitic infections or allergic asthma [28]. In the tumor microenvironment, MR and Mgl-1/2 have been documented to act as recognition molecules for glycosylated antigens on cancer cells. MR and Mgl-1/2 recognize specifically highly glycosylated molecules such as mucins present in the tumor microenvironment [30], leading to the expression of the immunoregulatory cytokine IL-10 that favors the attraction of Treg cells [25]. Indeed, Mgl-1/2 is predominantly expressed by TAMs from human ovarian carcinoma [31], on lung metastasis produced by mouse metastatic ovarian tumor cells [32], and also they have been detected after challenging tumor conditioned medium [33].

Chitinase 3-like 3 (Ym1) is a member of the mammalian chitinase family that also includes Ym2 and BRP-39 in mice, and YKL-40 in humans [34]. Increased expression of these proteins has been associated with inflammatory diseases, in particular with allergic asthma, with the induction of alternative activation of macrophages, and with progression of cancer [35–39].

Fizz-1, also known as resistin-like molecule *α* (RELM-*α*), plays a key role in the regulation of cell growth/proliferation and differentiation. Initially described in lung allergic inflammation [40], it is highly expressed in bleomycin-induced lung fibrosis and after IL-4 or IL-13 activation [41, 42]. Fizz-1 exhibits multiple functions including cell proliferation, angiogenesis, and inflammation [43], and its expression is an indicator of M2 polarization [21, 39]. 

Stabilin-1, a type-1 transmembrane receptor that mediates clearance of “unwanted self” components, is another marker for M2 macrophages that has been found to be expressed by TAM in several murine tumor models (e.g., B16 melanoma, pancreatic insulinoma, breast carcinoma) [19, 44]. Stabilin-1 mediates internalization of extracellular secreted protein acidic and rich in cysteine (SPARC), regulating its concentration and thereby promoting extracellular matrix remodeling, angiogenesis, and tumor progression [45]. 

## 4. Role of TAMs in Tumors

TAMs derive from circulating monocytic precursors previously recruited to the tumor region in response to the chemokines and cytokines secreted by cancer cells. In the tumor mass, TAMs exert immunosuppressive functions through the release of anti-inflammatory cytokines, modulate the tumor microenvironment producing survival/growth factors (e.g., vascular endothelial growth factor, VEGF), and facilitate the progression of tumors via proangiogenic factors release [25, 26].

### 4.1. TAMs and Immunosuppression

A relevant function of TAMs is to diminish the effective antitumor immune response. Several cytokines and proteases derived from TAMs, such as transforming growth factor (TGF)-**β*,* IL-10 and Arg-1, make a significant contribution to the immunosuppressive condition [46–48]. 

TGF-*β* inhibits the antitumor response through different mechanisms including (i) inhibition of the cytolytic activity of NK cells [48, 49], (ii) differentiation of CD4^+^ T cells into Th2 cells [50], (iii) inhibition of the CD8^+^ T cells antitumoral activity [48], and (iv) maintenance of Treg cell differentiation [48]. 

IL-10 promotes the immune evasion impeding the production of IL-12, a cytokine known to stimulate both the proliferation and cytotoxicity of T and NK cells [51], as well as the release of the cytokine IFN-*γ*, which is the main factor that stimulates naive T-cell differentiation [52]. Additionally, it has been reported that IL-10 decreases the ability of epidermal antigen presenting cells (APCs) to present tumor-associated antigens, therefore interfering the induction of antitumor immune responses [51].

High Arg-1 activity has been described in TAMs from 3LL murine lung carcinoma [22], human papillomavirus E6/E7-expressing murine tumors [53], and CD11b+/CD14− myeloid cells from renal carcinoma patients [54]. Elevated Arg-1 expression might promote tumor growth via several mechanisms including downregulation of NO-mediated tumor cytotoxicity [55], increasing cellular proliferation through its participation in polyamine and proline synthesis, dysregulating the T cell receptor (TCR) signaling and subsequently inducing CD8^+^ T cell unresponsiveness [47] and enhancing the capacity of myeloid suppressor cells to inhibit T cell proliferation [22].

Finally, TAMs release chemokines that play fundamental roles in immunosuppression. Ccl13, Ccl18 (human only), Ccl22, and to a lesser extent Ccl17 are important chemoattractant for immune-inhibitory cells, such as Treg, which might inhibit antitumor immunity resulting in tumor growth and decreased patient survival [16]. Moreover, Ccl2 and Ccl5 suppress the T-cell responses [16].

### 4.2. TAMs and Angiogenesis

Accumulating evidence indicates that TAMs exert a critical function in regulating angiogenesis, the process by which new blood vessels sprout from the existing vasculature. TAMs depletion studies in mice showed reduction of blood vessel density in the tumor tissue [56] and numerous correlations between increased TAM numbers and high vascular grades have been reported for many tumor types [57–62].

TAMs support tumor cell invasion by secreting a broad repertoire of molecules, including growth factors, cytokines, proteases, and chemokines. For instance, TAMs release potent proangiogenic cytokines such as IL-8 (or Cxcl8) and growth factors as vascular endothelial growth factor (VEGF), platelet-derived growth factor (PDGF), and TGF-**β**, which have been reported to promote angiogenesis in tumors such as gliomas, squamous cell carcinomas of the esophagus, and breast, bladder, and prostate carcinomas [59–63]. Importantly, they also release chemokines like Ccl2, Ccl5, Cxcl9, and Cxcl16 that contribute to angiogenesis [64]. Moreover, TAM-derived proteases, such as matrix metalloproteases (MMP-1, MMP-2, MMP-3, MMP-9, and MMP-12) are also beneficial to angiogenesis [65–67]. 

Additionally, TAMs have been found to accumulate in hypoxic regions of human and experimental tumors (including human endometrial, breast, prostate, and ovarian carcinomas) [68]. TAMs respond to the hypoxic microenvironment by upregulating the hypoxia-inducible transcription factors HIF-1 and HIF-2 that induce the expression of proangiogenic genes, such as VEGF, Cxcl8, and Cxcl12. 

### 4.3. TAMs and Tumor Growth

Macrophage depletion studies have proven that TAMs are essential for tumor growth [69] and TAMs infiltration has been observed in several tumors such as breast cancer, endometrial cancer, and renal cell cancer [70], demonstrating a positive correlation between proliferation of tumor cells and TAMs infiltration. Several molecules/factors secreted by TAMs such as MMP-9, IL-23, IL-10 facilitate tumor cell proliferation thereby limiting the cytotoxicity of the microenvironment. 

### 4.4. TAMs and Metastasis

Another process in which TAMs have been involved is in the regulation of metastasis. Indeed, a correspondence between the number of macrophages in metastatic sites and the metastatic potential of the tumor has been observed [71], and systemic depletion of macrophages results in reduced formation of lung metastases [72]. These findings are in line with clinical studies showing that increased numbers of macrophages in regional lymph node metastases correlates with poor patient survival [73].

TAMs appear to influence the microenvironment to facilitate migration of tumor cells [74, 75] by the release of MMPs, for example, MMP-2, MMP-7, and MMP-9. Those MMPs contribute to transform the proteins of the extracellular matrix and induce the expression of lymphatic endothelial growth factor (VEGF-C), events that promote dissemination of tumor cells by stimulating the formation of lymphatic vessels in tumors [76].

## 5. Tumor Suppressors and Immune System

Tumor suppressor genes act as sensors of multiple forms of cellular stress, being regulated to induce cell-cycle arrest, senescence, or apoptosis. Nevertheless, in last decades, a growing body of evidence indicates that tumor suppressors play a key role in the modulation of innate immune system, a function more relevant even than their activity as cancer inhibitors. 

For instance, innate immune response in the metazoan *Caenorhabditis elegans* has been described to be dependent on p53 function [77]. A clear function in antiviral defense has been reported for the tumor suppressors p53, the promyelocytic leukemia (PML) protein, and the alternative reading frame (ARF) [78–82]. Activation of PML, p53, and ARF has been described after IFN treatment, expression of viral proteins and viral infection [79, 82–87].

In addition, a molecular link between p53, ARF, retinoblastoma protein (Rb), and Toll-like receptors (TLRs) has been shown [87–89] and recent studies have demonstrated that ARF regulates inflammatory response [87].

### 5.1. The Retinoblastoma Protein Rb

Rb was identified as the protein responsible for the congenital tumor retinoblastoma [90] and plays pivotal roles in cell cycle control, differentiation, and inhibition of oncogenic transformation. Rb regulates cellular proliferation by directly binding to E2F transcription factors [91, 92], a family of transcription factors that regulates cellular proliferation, growth, and differentiation. 

Furthermore, additional functions of Rb in the control of immune response have been described including a novel role in viral infection surveillance. Thus Rb is required for the activation of the NF-*κ*B pathway in response to virus infection [93]. In addition, a recent report has shown that Rb positively regulates expression of TLR3, the sensing receptor for viral double-stranded RNA [89]. The mechanism involves modulation of the transcription factor E2F1, which directly binds to the proximal promoter of TLR3.

### 5.2. The Promyelocytic Leukemia (PML) Protein

The PML gene was originally identified in acute promyelocytic leukemia (APL), being implicated in numerous cellular functions including oncogenesis, DNA damage, senescence, apoptosis, and protein degradation. In addition, accumulating reports have also demonstrated the role of PML in host antiviral defense [78]. PML functions as the organizer of PML nuclear bodies (NBs) that contains some proteins recruited in a transient manner and two permanent NB-associated proteins, the IFN-stimulated gene product Speckled protein of 100 kDa (Sp100) and death-associated dead protein (Daxx) [94]. PML is induced by IFN leading to a marked increase in the expression of several PML isoforms (PMLI-PMLVII) and NBs. PML confers resistance to numerous virus including foamy virus (HFV), vesicular stomatitis virus (VSV), influenza virus, poliovirus, rabies virus, lymphocytic choriomeningitis virus (LCMV), and encephalomyocarditis virus [78, 95–99]. Interestingly, viruses inhibited by PML have developed various strategies to counteract the antiviral defense mechanisms by altering PML expression and/or localization on nuclear bodies [100].

### 5.3. Tumor Suppressor p53

The tumor suppressor p53 also known as “the guardian of genome” is activated in response to several types of cellular stress, including DNA damage and oncogene expression. Under normal conditions, p53 is maintained at very low levels through regulation by murine double minute 2 (Mdm2) protein. Mdm2 inhibits p53 transactivation and prompts p53 for proteasomal degradation by promoting its ubiquitination [101, 102]. However, in response to cellular stress, such as DNA damage, heat shock, or hypoxia, p53 levels rise as a consequence of activation of the tumor suppressor ARF that binds to Mdm2 and inhibit the ubiquitination, nuclear export, and subsequent degradation of p53 [103]. 

p53 has been implicated in multiple functions that play key roles in health and disease, including ribosome biogenesis, control of aging, cell cycle arrest, and apoptosis, having a clear importance in tumor suppression [104]. Interestingly, several lines of evidence also indicate that p53 may have a broader function in antiviral defense. Activation of p53 by IFN has been reported [80, 81] and p53-deficient mice are more permissive to viral infection [82]. This p53-mediated protection against viral infection is related with an induction of apoptosis, which is associated with reduced viral replication [82]. Moreover, in addition to activation of p53 by IFN, several genes involved in innate immunity have been described to be p53 direct transcriptional targets. IFN regulatory factors (IRFs) such as IRF-9 and IRF-5 have been described to be modulated by p53 [80]. Several mechanisms have been proposed for regulation of IRF-9 by p53 including upregulation at the transcriptional level [80], transactivation in response to influenza virus infection [105], and direct p53-IRF9 protein interaction upon Hepatitis C virus (HCV) infection [106]. Regarding IRF-5, an increase in IRF-5 levels in cancer cell lines has been shown through p53 binding and transactivation of the IRF-5 promoter [107].

Pattern recognition receptors such as TLR3 have also been reported to be regulated by p53 [88]. TLR3 plays a major role in the recognition of virus infection leading to the induction of the IFN pathway [108]. p53 activates TLR3 transcription by binding to the p53 consensus site in the TLR3 promoter. Moreover, TLR3 expression was downregulated in liver and intestine of p53^−/−^ mice and HCT116 p53^−/−^ cells, leading to a dysfunction in both NF-*κ*B and IRF-3 signaling pathways [88]. Upregulation of TLR3 activity by p53 may also be responsible for the activation of interferon-stimulated gene 15 (ISG15). ISG15 is strongly induced by type I interferons and displays antiviral activity. Although a functional p53 binding site adjacent to the core ISRE site of ISG15 has been reported, upregulation of ISG15 has been observed after dsRNA stimulation rather than in response to IFN treatment or virus infection, suggesting that the observed effects on ISG15 could be mediated through p53-dependent upregulation of TLR3 activity [109].

Finally, important proinflammatory chemokines such as monocyte-chemoattractant protein (MCP)-1 have also been reported to be transcriptionally regulated by p53 [110]. MCP-1, also known as Ccl2, triggers the infiltration and activation of cells of the monocyte-macrophage lineage and has been linked with antitumor immunity [111] and cervical cancer [112]. 

Thus, in addition to the well-established function of p53 as a tumor suppressor through regulation of apoptosis or cell cycle, p53 exerts essential roles in the expression of key molecules of the innate immune response. Indeed, the loss of p53 function during carcinogenesis might affect the recognition of tumor cells by the immune system through interfering with inflammatory mediators expression. 

### 5.4. The Tumor Suppressor ARF

Tumor suppressor ARF (p14ARF in human, p19ARF in mouse) is among the most frequent genes mutated in human cancer [113]. ARF is encoded by the *INK4a/ARF *locus (Cdkn2a) that generates two unrelated proteins, the cyclin-dependent kinase inhibitor p16INK4a and ARF, which, respectively, regulate the activity of Rb and the p53 transcription factor [114, 115]. ARF activates p53 by sequestering Mdm2, an E3 ubiquitin ligase, to the nucleolus, thereby inhibiting the Mdm2-mediated proteasomal degradation of p53. p53 subsequently activates p21 (CIP1/WAF1), which inhibits the cell cycle [103, 116]. Although tumor suppressor activity of ARF was initially attributed to p53 regulation, several p53-independent actions for ARF have been described [117]. Thus, ARF inhibits ribosomal RNA processing [118] and transcriptional factors that induce proliferation such as E2F1, Myc, and Forkhead box M1 (Foxm1b) [119–121]. In addition, ARF interacts with the protein related with cell proliferation nucleophosmin (NPM) [122].

Recent studies have shown that the tumor suppressor ARF is more than a simple tumor suppressor and acts a general sensor for different situation of cellular stress. In this context, a regulatory network between Heat shock protein 70 (Hsp70), ARF, and *β*-catenin has been shown after oxidative stress that leads to the induction of apoptosis [123]. ARF deficiency has been reported to aggravate atherosclerosis through the reduction of macrophage and vascular smooth muscle cell apoptosis [124]. ARF is also expressed transiently during mouse male germ cell and eye development and its inactivation compromises spermatogenesis as mice age and leads to aberrant postnatal proliferation of cells in the vitreous of the eye, resulting in blindness [125]. 

 Furthermore, ARF plays an important role in the regulation of innate immunity and inflammatory processes. Several reports have described an antiviral action of ARF as well as its activation after the expression of viral proteins, viral infection, or type I IFN treatment [79, 85–87]. Indeed, the analysis of the ARF promoter revealed the presence of IFN response elements such as IRF-3 and interferon-sensitive response element (ISRE). The protective effect of ARF against viral infection seems to be a general feature for IFN-sensitive viruses as demonstrated the studies using VSV and Sindbis virus or vaccinia virus (VV) [79]. Mechanism involved in this protection is due, at least in part, to interaction with NPM and activation of the double-stranded RNA-dependent protein kinase, PKR [79].

**Figure 2:**
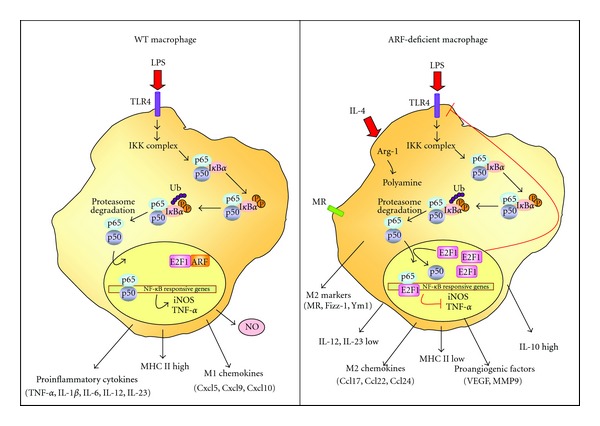
Alternative activation in ARF-deficient macrophages. In WT macrophages, a balance between M1 and M2 phenotype is established, depending on stimuli. Inflammatory stimuli induce NF-*κ*B signaling pathways through the phosphorylation and subsequent ubiquitin-dependent degradation of I*κ*B*α* by the 26S proteasome. Then, NF-*κ*B translocates to the nucleus inducing target gene expression. ARF present in the nucleus displays physical and functional interaction with E2F1 resulting in destabilization of E2F1 protein and activation of NF-*κ*B. However, ARF-deficient macrophages establish an immunosuppressive and tolerant microenvironment via impairment of M1 signals. When NF-*κ*B translocates to the nucleus, excessive E2F1 inhibits NF-*κ*B by binding to its subunit p65 in competition with the heterodimeric partner p50. Moreover, excessive E2F1 may inhibit transcriptional expression of TLRs. This leads to secretion of M2 chemokines Ccl17 and Ccl22, release of the anti-inflammatory cytokine IL-10, and stimulation of angiogenesis through expression of VEGF and MMP-9.

Interestingly, it has been recently described that ARF is a critical modulator of the inflammatory response and macrophage activation. It has also been reported a molecular link between ARF and TLRs [87]. Additionally, a manuscript by Herranz et al. [126] suggests that ARF might modulate the M1/M2 polarization and functional plasticity of macrophages (Figure 2).

Mice lacking the ARF gene are resistant to LPS-endotoxic shock, and a significant reduction of leukocyte recruitment in a model of thioglicollate-induced peritonitis was also reported [87]. Moreover, ARF-deficient macrophages present an impaired ability to develop proinflammatory properties showing a relevant downregulation of genes involved in M1 macrophage phenotype and inhibition of the antimicrobial and antitumoral responses, including expression of proinflammatory cytokines (TNF-*α*, IL-1*β*), chemokines (Cxcl10, Cxcl1, Ccl4), and inflammatory mediators (iNOS/PGE_2_). Mechanisms involved in this inhibitory effect have not been fully explored, although a decrease in NF-*κ*B and Mitogen-activated protein kinases (MAPK) activation have been described in ARF−/− macrophages after stimulation with LPS [87], as well as inhibition of I*κ*B degradation in ARF-deficient macrophages stimulated with VSV [79]. Furthermore, an increase of the transcription factor E2F1 at basal state and after LPS-stimulation has been shown in absence of ARF [87]. E2F1 has been proposed to be an anti-inflammatory and immunosuppressive transcription factor since it represses NF-*κ*B-dependent inflammatory signaling [127, 128]. Therefore, it has been proposed that in normal cells, ARF interacts with E2F1, resulting in destabilization of E2F1 protein and activation of NF-*κ*B. In contrast, in the absence of ARF, E2F1 is overexpressed, and although NF-*κ*B translocates to the nucleus, excessive E2F1 inhibits its activity by binding to p65 and thereby suppressing NF-*κ*B-dependent genes (iNOS, COX-2, chemokines, etc.). 

Loss of ARF gene can abrogate tumor surveillance mechanisms and increase cancer susceptibility. Indeed, mice lacking p19ARF are highly prone to tumor development [129, 130] and deletion of ARF has been described in a variety of malignancies, including glioblastoma, melanoma, pancreatic adenocarcinoma, and non-small-cell lung cancer. Interestingly, M2-polarized TAMs have been demonstrated to be associated with poor prognosis and progression in many of these tumors. 

However, the link between ARF and immune response in the tumoral context remains an open question. A manuscript by Herranz et al. [126] goes deep into the immune role of ARF, evaluating its possible contribution to the M1/M2 polarization of macrophages. In this study, authors demonstrate that ARF deficiency switches macrophages to a M2-like phenotype. Thus, in addition to downregulation of proinflammatory mediators, typical hallmarks of an anti-inflammatory M2-activation state were increased in both resting and IL-4-treated ARF−/− macrophages, as exemplified by the upregulation of Arg-1, Fizz-1 and Ym1. Moreover, the cytokine/chemokine pattern induced by IL-4 stimulation appeared upregulated in macrophages isolated from ARF−/− mice showing an M2-phenotype with higher levels of IL-10, Ccl22, Ccl5, Ccr3, or Ccr5. Together with these M2 markers, important proangiogenic factors such as VEGF and MMP-9 were also increased, suggesting the potential protumoral action of ARF−/− macrophages. Consistent with this notion, recent studies have demonstrated a role for ARF in suppressing tumor angiogenesis via modulation of VEGF expression [131] and the activity of its transactivator HIF [132]. Notably, ARF has also been described to inhibit angiogenesis by up-regulating the expression of TIMP3, an inhibitor of MMPs activity [133]. 

Taken together, it is tempting to speculate that ARF has a profound influence in regulating the polarization of macrophages. Thus, ARF deficiency might modify the immune tumor microenvironment as a result of the induction of multiple activities including (a) immune suppression, through the production of the anti-inflammatory cytokine IL-10 and the secretion of Ccl22 and Ccl2 that attracts Tregs, (b) stimulation of angiogenesis through expression of VEGF and MMP-9, and (c) induction of matrix remodeling through the production of MMP-9, Fizz-1, and Ym1. 

## 6. Conclusions 

More and more evidence indicates that tumor suppressors play an essential role in host immunity, placing them as general sensors and modulators of innate immune response. Mechanisms involved in this process remain unclear, although transcriptional regulation of several inflammatory mediators has been reported. In addition, M1/M2 polarization and functional plasticity of macrophages have been shown to be modulated by tumor suppressors. These studies reveal positive effects of tumor suppressors on cancer immunosurveillance that will pave the way for new targeted therapies.

Therefore, identification of the role of tumor suppressors in the pathways responsible for the skewing of macrophage function as well as in the regulation of inflammatory mediators will remain an important area of investigation in the years to come.
